# The association of motor reserve and clinical progression in Parkinson’s disease

**DOI:** 10.1016/j.nicl.2024.103704

**Published:** 2024-11-09

**Authors:** Xueqin Bai, Shiwei Zhang, Qiuyue Li, Tao Guo, Xiaojun Guan, Andan Qian, Shuangli Chen, Ronghui Zhou, Yitong Cheng, Haoxin Chen, Zhaoke Gou, Chenglong Xie, Zhen Wang, Minming Zhang, Xiangwu Zheng, Meihao Wang

**Affiliations:** a Department of Radiology, The First Affiliated Hospital of Wenzhou Medical University, 325000 Wenzhou, China; b Department of Radiology, The Second Affiliated Hospital, Zhejiang University School of Medicine, 310009 Hangzhou, China; c Department of Neurology, The First Affiliated Hospital of Wenzhou Medical University, 325000 Wenzhou, China; d The First Affiliated Hospital of Wenzhou Medical University and Key Laboratory of Intelligent Medical Imaging of Wenzhou, Wenzhou, China

**Keywords:** Parkinson’s disease, Motor reserve, Clinical progression, Cognition

## Abstract

**Objective:**

To explore the association of motor reserve (MR) and clinical progression in Parkinson’s disease.

**Methods:**

This longitudinal study using data from the Parkinson’s progression markers initiative. Patients with de novo PD who underwent dopamine transporter scans at baseline and finished at least five years clinical follow-up assessments (including motor, cognitive, and non-motor symptoms) were included. The individual MR of PD patients were estimated based on initial motor deficits and striatal dopamine depletion using a residual model. Linear mixed-effects models (LME) were performed to examine the associations of baseline MR and clinical progression.

**Results:**

A total of 303 de novo PD patients were included and the mean follow-up time was 8.95 years. Results of LME models revealed that the baseline MR was associated with motor, cognitive, and non-motor symptoms in PD patients. There was a significant interaction between MR and disease duration for longitudinal changes in motor (*p* < 0.001), cognitive (*p* = 0.028) and depression symptoms (*p* = 0.014). PD patients with lower MR had a more rapid progression to postural instability and cognitive impairment compared with those with higher MR (*p* = 0.002 and *p* = 0.001, respectively).

**Conclusions:**

The baseline MR of PD patients were associated with motor and non-motor symptoms and can predicted disease prognosis, suggesting that the initial MR in PD would be associated with the individual’s capacity to cope with neurodegenerative process as well as comprehensive prognosis.

## 1 Introduction

Parkinson’s disease (PD) is one of the most common neurodegenerative diseases and follows a progressive course (Bloem et al., 2021). The clinical presentations of PD are complex; with characteristic motor symptoms (bradykinesia, rigidity and resting tremor, etc.) and variable non-motor features (cognitive impairment, depression, etc.). As the disease progresses, motor symptoms in PD patients increasingly aggravated (Simuni, 2018), and cognitive function progressively declined (Hely et al., 2008), the probability of developing disabling movement disorders and dementia rises significantly, which puts great burden on the family and society. It is widely acknowledged that the prognosis of PD is heterogeneous, with different patients exhibiting varying rates of disease progression (Fereshtehnejad, 2015, De Pablo-Fernandez et al., 2019). Therefore, identifying prognostic factors for PD is of great significance for predicting disease progression and guiding clinical management.

The concept of reserve was introduced to explain the discrepancy between the degree of observed pathological changes in the brain and clinical manifestations. Similar with cognitive reserve, the concept of motor reserve (MR) has been proposed to explain the presence of individual heterogeneity in the severity Parkinsonism motor symptom, despite a similar pathological injury (Chung, 2020, Chung et al., 2020). The core pathological mechanism of PD is the loss of dopaminergic neurons in the substantia nigra (Braak, et al., 2003) and the secondary reduction of dopamine in the basal ganglia (Bloem et al., 2021), which can be reflected by dopamine transporter (DAT) scans (Poewe and Scherfler, 2003). Therefore, the MR in PD patients can be evaluated with the discrepancy between the severity of motor symptoms and the extent of DAT deficit. Individuals with greater MR may cope better with brain damage than those with lower MR. Previous studies on Alzheimer’s disease (AD) showed the cognitive reserve was associated with cognitive performance and individuals with different levels of reserve present different rates of cognitive decline (van Loenhoud, 2019, Stern, 2012). Similarly, MR in PD is thought to influence both clinical symptoms and disease progression. However, there is little evidence of the impact of the initial MR on clinical progression in PD. A Previous study reported that the great MR was associated with a lower risk for levodopa-induced dyskinesia and freezing of gait (Chung, 2020). Recently, a study further investigated the association between MR and cognitive function and found that the MR was positively correlated with the global cognitive function and the verbal memory function domain in PD patients (Chung, 2022). PD patients with higher MR were also found to have a lower risk of conversion to dementia (Chung, 2022). However; the influence of initial MR on the composite clinical progression, including motor and non-motor symptoms, remains unclear.

In this study, we estimate the MR for each patient and investigated whether initial MR plays a role in the progression of motor, cognitive and non-motor symptoms in a longitudinal PD cohort. We hypothesized that the initial MR levels in PD patients are associated with clinical progression.

## 2 Methods

### 2.1 Subjects

Participants data in this study were obtained from the Parkinson’s Progression Markers Initiative (PPMI) database on November 1, 2023. PPMI is an ongoing prospective, longitudinal, multi-center study aimed at identifying clinical, imaging, genetic, and biospecimen biomarkers of PD progression (Marek, 2018). PD patients underwent motor function evaluations at baseline, every 3 months in the first year, every 6 months in the next 4 years, and every 12 months afterward, and receive evaluation of cognitive and non-motor function at baseline and annually thereafter. Study protocols and manuals are available on the PPMI website (https://www.ppmi-info.org). At baseline, PD participants were required to (1) have a recent idiopathic PD diagnosis, (2) be untreated for PD, (3) have DAT deficit. The inclusion criteria in this study included the following:(1) baseline Hoehn and Yahr (HY) stage < 3; (2) PD patients without dementia at baseline (Montreal Cognitive Assessment (MoCA) > 21) (Dalrymple-Alford, 2010); (3) patients finished at least five years follow-up.

### 2.2 Baseline and follow-up clinical evaluation

Demographic information, including age, sex, years of education and disease durations were obtained at baseline. The motor, cognitive and non-motor characteristics were assessed using the Movement Disorder Society Unified Parkinson’s Disease Rating Scale (MDS-UPDRS) (Parts I through III) (Goetz, 2007); HY stage, MoCA, the Geriatric Depression Scale (GDS), State-trait anxiety inventory(STAI), Rapid eye movement Sleep Behavior Disorder Questionnaire (REM), the Scale for Outcomes in Parkinson’s disease-Autonomic (SCOPA), and the Modified Schwab and England Activities of Daily Living (ADL). These assessments were recorded both at baseline and follow-up visits. Besides, detailed neuropsychological assessments were also collected, including visuospatial function (Benton Judgment of Line Orientation Test, BJLOT) (Qualls et al., 2000), processing speed-attention (Symbol Digit Modalities Test, SDMT) (Forn, 2009), verbal learning and memory (Hopkins Verbal Learning Test for delayed recall, HVLT) (Shapiro et al., 1999), semantic fluency (Semantic Verbal Fluency Test, SFT) (Shao et al., 2014), and working memory capacity (letter-number sequencing, LNS) (Gladsjo, 1999). For PD patients initiating PD medications after enrollment, the levodopa equivalent daily dose (LEDD) was recorded at follow-up visits.

### 2.3 DAT imaging and processing

All patients received DAT ^123^I-ioflupane single photon emission computed tomography (SPECT) imaging at baseline. The DAT images were acquired at PPMI imaging centers according to the PPMI imaging protocol and sent to Institute for Neurodegenerative Disorders for processing and calculation of striatal binding ratios (SBR) (Tinaz, 2018). Striatal regions of interest (ROI) were placed on the target regions (bilateral caudate and putamen) and reference region (occipital cortex). Then, mean standardized uptake value of each ROI were extracted and used to calculate SBR for each striatal ROI (Tinaz, 2018). SBR was calculated as follows: SBR_target region_ = (target region / reference region) − 1. The mean SBR of bilateral putamen was calculated and used as DAT availability for subsequent analysis (Chung, 2020).

### 2.4 Estimation of motor reserve

The MR of each patient was estimated based on the baseline MDS-UPDRS III score and DAT availability in the putamen (Chung, 2020). We used the general linear model to predict the MDS-UPDRS III score by using age, sex, disease duration, and DAT availability in the putamen. Then, the residuals (differences between the actual value and the predicted value of MDS-UPDRS III score) in the general linear model were calculated and standardized as follows: motor reserve = standardized value of (MDS-UPDRS III_predicted_ − MDS-UPDRS III_observed_) (Lee, 2019). A greater standardized value means the higher MR; indicating the participant had a lower MDS-UPDRS III score than the predicted score. In addition, to estimate the different influence of MR on clinical symptoms in PD patients, we set the cutoff as 0.5 and −0.5 of MR respectively as high and low MR groups (Chung, 2020).

### 2.5 Statistical analyses

The statistical analyses in this study were conducted using the IBM SPSS statistical software (version 26.0) and the linear mixed-effects (LME) models were conducted in Matlab (R2019a, lme4 package). Demographic and clinical data were analyzed using one-way analysis of variance (ANOVA) or Kruskal-Wallis test among PD patients with different levels of MR. Differences in sex distribution were compared using chi-square test.

LME models were employed to investigate the longitudinal changes in clinical symptoms of PD patients as the disease progresses, as well as the association between the baseline MR levels and clinical symptom progression. The clinical symptoms analyzed included motor symptoms (MDS-UPDRS (Parts I through III)), cognitive symptoms (MoCA,visuospatial function (BJLOT), processing speed-attention (SDMT), verbal learning and memory (HVLT), semantic fluency, SFT), and working memory capacity (LNS)), non-motor symptoms (GDS, STAI, REM, SCOPA) and daily living function (ADL). For the longitudinal progression of clinical symptoms, each clinical symptom was included in the model as a dependent variable, with disease duration as the independent variable. Confounding variables such as age, sex, education, and LEDD were adjusted as fixed effects in these LME models, with random intercept and slope for each participant. To assess the association between the baseline MR and clinical symptoms, separate LME models were built for each clinical score, with baseline MR as the independent variable and each clinical score as the dependent variable. Confounding variables, including age, sex, education, and LEDD, were adjusted as fixed effects in these models, with random intercepts and slopes for each participant.

Furthermore, LME models were applied to explore whether baseline MR levels could predict clinical progression in PD. In these models, baseline MR, time-varying disease duration, and their interaction were treated as fixed effects, with the intercept treated as a random effect. Clinical scores were separately used as dependent variables, with age, sex, education, and LEDD included as covariates. The specific equations for the LME models are provided in the supplementary materials.

The Kaplan-Meier survival analyses were performed to compare the cumulative probability of postural instability and cognitive progression between high MR and low MR groups during the follow-up period. The outcome event for postural instability was defined as reaching HY stage ≥ 3 (Lin, 2023) within five years of follow-up, while cognitive progression was defined as the development of cognitive impairment (mild cognitive impairment (MCI) or dementia) in PD patients with normal cognition during five years follow-up. Global cognitive function was assessed using MoCA. Cognitive status was categorized based on the criteria used from previous studies: normal cognition (MoCA ≥ 26), MCI (22 ≤ MoCA ≤ 25), dementia (MoCA < 22). All analyses of LME models were corrected for multiple comparisons by using false discovery rate (FDR) correction. The significance threshold was set at two-tailed *p* < 0.05.

## 3 Results

### 3.1 Demographics and clinical characteristics

A total of 303 PD patients were included in this study, and all baseline demographic are illustrated in Table 1. All patients completed at least five years follow-up, with a mean follow-up time of 8.95 years. The longitudinal changes in clinical characteristics are shown in Table 2. To provide a clearer summary of clinical progression, only the baseline and the last visit data were showed. The longitudinal analyses revealed significant progression in all motor and non-motor measures over time in PD patients, although the magnitude of progression varied. In LME models, the severity of MDS-UPDRS Parts I, II, III, REM, GDS, STAI and SCOPA were increased by approximately 0.53 (95 % CI, 0.44 to 0.63), 0.96 (95 % CI, 0.84 to 1.07), 2.04 (95 % CI, 1.80 to 2.27), 0.12 (95 % CI, 0.07 to 0.18), 0.10 (95 % CI, 0.05 to 0.15), 0.41(95 % CI, 0.08 to 0.73), 0.39 (95 % CI, 0.27 to 0.50) units per year, and MoCA and ADL score decreased by approximately 0.07 (95 % CI, − 0.14 to −0.02) and 1.50 (95 % CI, −1.73 to −1.27) units per year. For detailed cognitive domains, all the cognitive domains showed progression over time (SDMT (estimate −0.55, 95 % CI −0.75 to −0.35), BJLOT (estimate −0.04, 95 % CI −0.08 to 0), LNS (estimate −0.10, 95 % CI −0.15 to −0.05), HVLT (estimate −0.13, 95 % CI −0.19 to −0.08), SFT (estimate −0.19, 95 % CI −0.29 to −0.09)). We additionally added the baseline clinical score as a covariate in the LME model and the results remained consistent with those obtained previously (see Supplementary materials).

**Table 1:** The baseline demographic and clinical data.

**Characteristics**	**PD patients**	**Baseline MR levels**			**P value**
		**High MR (N = 100)**	**Moderate MR (N = 116)**	**Low MR (N = 87)**	
**Age (y)**	**60.79** ± 9.74	**60.92** ± **10.09**	**60.16** ± **10.53**	**61.49** ± **8.18**	**0.626**
**Gender (M/F)**	**200/103**	**65/35**	**78/38**	**57/30**	**0.362^c^**
**Education (y)**	**15.76 ± 2.91**	**15.67 ± 2.96**	**15.78 ± 3.15**	**15.84 ± 2.53**	**0.922**
**Disease duration (y)**	**0.58 ± 0.59**	**0.67 ± 0.67**	**0.55 ± 0.53**	**0.58 ± 0.57**	**0.857**
**Hoehn and Yahr stage, median (range)**	**2(1**–**2)**	**1(1**–**2)**	**2(1**–**2)**	**2(1**–**2)**	**0.286^b^**
**MDS-UPDRS I**	**5.20 ± 3.77**	**4.07 ± 3.20**	**5.41 ± 3.91**	**6.23 ± 3.90**	< 0.001^a**b***^
**MDS-UPDRS II**	**5.48 ± 4.00**	**3.81 ± 3.06**	**5.84 ± 3.84**	**6.92 ± 4.51**	< 0.001^a***b***c*^
**MDS-UPDRS III**	**19.94 ± 8.48**	**12.01 ± 3.63**	**19.09 ± 3.88**	**30.17 ± 6.11**	<0.001^a***b***c***^
**ADL**	**93.60 ± 5.81**	**95.85 ± 4.98**	**93.41 ± 5.77**	**91.26 ± 5.82**	<0.001^a**b***c**^
**REM**	**3.95 ± 2.56**	**3.67 ± 2.29**	**3.91 ± 2.51**	**4.34 ± 2.88**	**0.192**
**GDS**	**2.37 ± 2.47**	**1.88 ± 2.09**	**2.25 ± 2.31**	**3.09 ± 2.90**	0.003^b***c*^
**STAI**	**65.27 ± 18.01**	**60.50 ± 15.71**	**65.27 ± 15.77**	**70.75 ± 21.56**	<0.001^a*b***c*^
**SCOPA**	**9.34 ± 6.30**	**8.09 ± 5.751**	**8.83 ± 5.534**	**11.43 ± 7.319**	<0.001^b***,c**^
**MoCA**	**27.32 ± 2.02**	**27.68 ± 1.885**	**27.28 ± 2.034**	**26.95 ± 2.102**	0.048^b*^
**BJLOT**	**13.01 ± 2.01**	**13.31 ± 1.926**	**13.03 ± 2.081**	**12.62 ± 1.984**	0.064^b*^
**HVLT**	**8.52 ± 2.48**	**8.84 ± 2.407**	**8.50 ± 2.458**	**8.20 ± 2.583**	**0.207**
**LNS**	**10.79 ± 2.60**	**11.17 ± 2.756**	**10.72 ± 2.338**	**10.45 ± 2.710**	**0.156**
**SDMT**	**42.29 ± 9.71**	**43.86 ± 9.291**	**42.65 ± 9.757**	**40.02 ± 9.819**	0.023^b**^
**SFT**	**21.19 ± 5.36**	**22.10 ± 5.722**	**20.97 ± 5.645**	**20.43 ± 4.363**	0.089^b*^

MDS-UPDRS: Movement Disorders Society Unifed Parkinson’s Disease Rating Scale; ADL: Modified Schwab and England Activities of Daily Living; REM:Rapid eye movement Sleep Behavior Disorder Questionnaire; GDS: Geriatric Depression Scale; STAI: State-trait anxiety inventory; SCOPA: Scale for Outcomes in Parkinson’s disease-Autonomic; MoCA: Montreal Cognitive Assessment; BJLOT: Benton Judgment of Line Orientation Test; HVLT: Hopkins Verbal Learning Test for delayed recall; LNS: letter-number sequencing. SDMT: Symbol Digit Modalities Test; SFT: Semantic Verbal Fluency Test.

**a the high MR group versus the moderate MR group;**

**b the high MR group versus the low MR group;**

**c the moderate MR group versus the low MR group;**

*P < 0.05.; **P < 0.01.; ***P < 0.001.

**Table 2:** The longitudinal changes of clinical characteristics.

Variable	Baseline	Last visit	Estimate	*p* value	*q* value
MDS-UPDRS I	5.20 ± 3.77	11.62 ± 7.16	0.53	**<0.001**	**<0.001**
MDS-UPDRS II	5.48 ± 4.00	14.06 ± 8.30	0.96	**<0.001**	**<0.001**
MDS-UPDRS III	19.94 ± 8.48	38.76 ± 15.86	2.04	**<0.001**	**<0.001**
ADL	93.60 ± 5.81	78.25 ± 16.72	−1.50	**<0.001**	**<0.001**
REM	3.95 ± 2.56	5.29 ± 3.51	0.12	**<0.001**	**<0.001**
GDS	2.37 ± 2.47	3.43 ± 3.17	0.10	**<0.001**	**<0.001**
STAI	65.27 ± 18.01	67.39 ± 21.12	0.41	**0.015**	**0.016**
SCOPA	9.34 ± 6.30	15.94 ± 8.00	0.39	**<0.001**	**<0.001**
MoCA	27.32 ± 2.02	25.91 ± 4.42	−0.08	**0.007**	**0.008**
BJLOT	13.01 ± 2.01	12.24 ± 2.62	−0.04	**0.043**	**0.043**
HVLT	8.52 ± 2.48	6.83 ± 3.61	−0.13	**<0.001**	**<0.001**
LNS	10.79 ± 2.60	9.02 ± 3.33	−0.10	**<0.001**	**<0.001**
SDMT	42.29 ± 9.71	35.45 ± 14.48	−0.55	**<0.001**	**<0.001**
SFT	21.19 ± 5.36	19.02 ± 6.39	−0.19	**<0.001**	**<0.001**

MDS-UPDRS: Movement Disorders Society Unifed Parkinson’s Disease Rating Scale; ADL: Modified Schwab and England Activities of Daily Living; REM:Rapid eye movement Sleep Behavior Disorder Questionnaire; GDS: Geriatric Depression Scale; STAI: State-trait anxiety inventory; SCOPA: Scale for Outcomes in Parkinson’s disease-Autonomic; MoCA: Montreal Cognitive Assessment; BJLOT: Benton Judgment of Line Orientation Test; HVLT: Hopkins Verbal Learning Test for delayed recall; LNS: letter-number sequencing. SDMT: Symbol Digit Modalities Test; SFT: Semantic Verbal Fluency Test.

### 3.2 Associations between MR values and clinical symptoms

The baseline MR values were associated with motor, non-motor, cognitive function in PD patients. In LME analyses, baseline MR was significantly associated with MDS-UPDRS Parts I, II, III, MoCA, LNS, SDMT, SFT, ADL, REM, GDS, STAI and SCOPA scores (Table 3). The higher baseline MR values were significantly associated with lower levels of MDS-UPDRS Parts I, II, III, REM, GDS, STAI and SCOPA scores. The higher baseline MR values were significantly associated with higher cognitive function (MoCA, β = 0.24, *p* = 0.018) and daily living function (ADL, β = 1.97, *p* < 0.001). For detailed cognitive analysis, the higher baseline MR values were significantly associated with higher processing speed-attention (SDMT, β = 1.35, *p* = 0.004), verbal working memory (LNS, β = 0.325, *p* = 0.010), language (SFT, β = 0.62, *p* = 0.033). There was no association between MR and the visuospatial function (BJLOT, *p* = 0.076) and verbal learning and memory function (HVLT, *p* = 0.073).

**Table 3:** Association between baseline motor reserve and motor, non-motor, cognitive, and functional scores in PD over time.

Variable	Estimate	*p* value	*q* value
MDS-UPDRS I	−0.57	**0.010**	**0.016**
MDS-UPDRS II	−1.27	**<0.001**	**<0.001**
MDS-UPDRS III	−6.93	**<0.001**	**<0.001**
ADL	1.97	**<0.001**	**<0.001**
REM	−0.36	**0.013**	**0.019**
GDS	−0.43	**0.002**	**0.005**
STAI	−3.34	**<0.001**	**0.002**
SCOPA	−1.04	**0.002**	**0.005**
MoCA	0.24	**0.018**	**0.023**
BJLOT	0.17	0.076	0.076
HVLT	0.22	0.073	0.076
LNS	0.33	**0.010**	**0.016**
SDMT	1.36	**0.004**	**0.008**
SFT	0.62	**0.033**	**0.038**

MDS-UPDRS: Movement Disorders Society Unifed Parkinson’s Disease Rating Scale; ADL: Modified Schwab and England Activities of Daily Living; REM:Rapid eye movement Sleep Behavior Disorder Questionnaire; GDS: Geriatric Depression Scale; STAI: State-trait anxiety inventory; SCOPA: Scale for Outcomes in Parkinson’s disease-Autonomic; MoCA: Montreal Cognitive Assessment; BJLOT: Benton Judgment of Line Orientation Test; HVLT: Hopkins Verbal Learning Test for delayed recall; LNS: letter-number sequencing. SDMT: Symbol Digit Modalities Test; SFT: Semantic Verbal Fluency Test.

### 3.3 Prediction of clinical progression using baseline MR values

LME models showed that baseline MR could predict longitudinal motor and cognitive progression in PD patients. There was a significant interaction between MR and disease duration for longitudinal changes in MDS-UPDRS III (*p* < 0.001), MoCA (*p* = 0.028), BJLOT (*p* = 0.041) and GDS (*p* = 0.014) scores after controlling for age, sex, education, and LEDD. Notably, our results of the interaction showed that PD patients with higher MR had a faster rate of motor progression. The PD patients with higher MR exhibited a slower rate of cognitive decline, although the statistical significance of the MoCA scores did not remain after FDR correction.

To account for the influence of motor symptoms, baseline MDS-UPDRS III scores were also included as a covariate, the LME results showed that baseline MR was also associated with the longitudinal changes in both motor and non-motor symptoms. Additionally, there was still a significant interaction between MR and disease duration for longitudinal changes in MDS-UPDRS III, MoCA, BJLOT and GDS (see Supplementary materials). See (Table 4).

**Table 4:** Summary of linear mixed-effects models of the initial MR on the longitudinal progression in clinical symptoms.

Variable	Estimate (95 %CI)	*p* value	q value
MDS-UPDRS I	0.009(−0.060–0.078)	0.797	0.797
MDS-UPDRS II	0.057(−0.037–0.150)	0.237	0.552
MDS-UPDRS III	0.366(0.181–0.551)	**< 0.001**	**0.002**
ADL	−0.085(−0.287–0.117)	0.410	0.574
REM	−0.010（-0.049–0.030）	0.632	0.737
GDS	0.050（0.010–0.895）	**0.014**	0.096
STAI	0.150（-0.081–0.381）	0.203	0.552
SCOPA	0.024（-0.049–0.097）	0.522	0.664
MoCA	0.0528（0.006–0.100）	**0.028**	0.130
BJLOT	0.028（0.001–0.056）	**0.041**	0.143
HVLT	0.016（-0.022–0.054）	0.410	0.574
LNS	0.014（-0.018–0.046）	0.397	0.574
SDMT	0.083（-0.066–0.233）	0.276	0.552
SFT	−0.011（-0.080–0.057）	0.744	0.797

MDS-UPDRS: Movement Disorders Society Unifed Parkinson’s Disease Rating Scale; ADL: Modified Schwab and England Activities of Daily Living; REM:Rapid eye movement Sleep Behavior Disorder Questionnaire; GDS: Geriatric Depression Scale; STAI: State-trait anxiety inventory; SCOPA: Scale for Outcomes in Parkinson’s disease-Autonomic; MoCA: Montreal Cognitive Assessment; BJLOT: Benton Judgment of Line Orientation Test; HVLT: Hopkins Verbal Learning Test for delayed recall; LNS: letter-number sequencing. SDMT: Symbol Digit Modalities Test; SFT: Semantic Verbal Fluency Test.

### 3.4 Prediction of baseline MR on progression to postural instability/cognitive transition

**Fig. 1:**
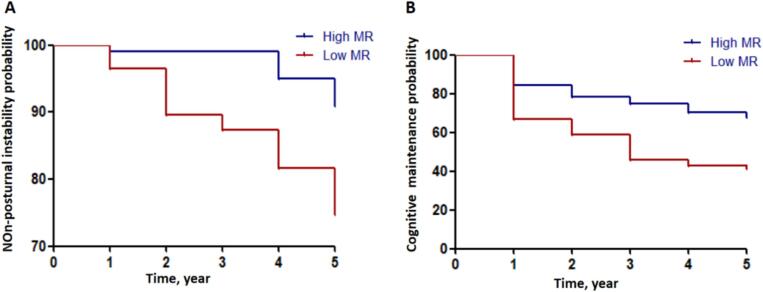
Baseline MR predicts progression to postural instability(A) and cognitive impairment (B) in PD.

Kaplan-Meir survival analyses demonstrated that patients with low MR had a more rapid progression to postural instability (HY ≥ 3) compared with those with high MR (*p* = 0.002; Fig. 1A). Additionally, during the follow-up period, we found that the lower baseline MR of PD patients with normal cognition, the higher the risk of cognitive progression (*p* = 0.001, Fig. 1B).

## 4 Discussion

In this longitudinal study, we investigated whether MR (estimated by MDS-UPDRS III and DAT) influence the progression of motor, cognitive and non-motor symptoms in PD patients. We found that the baseline MR levels are associated with motor, cognitive and non-motor function in PD patients over time. Besides, PD patients with different level of baseline MR values exhibit varying rates of motor and cognitive symptoms progression. PD patients with lower MR had higher risk of postural instability and progression to MCI or dementia.

In this study, we found that baseline MR levels are associated with motor, cognitive and non-motor function in PD patients. Our findings are consistent with our hypothesis, suggesting that patients with higher MR may cope better with brain damage than those with lower MR. Previous studies have shown that PD patients with lower MR exhibited higher motor scores (Chung, 2020), which was consistent with our findings. A recent study investigated the link between MR and cognition function and found that MR was positively associated with the global cognitive function and the verbal memory domain (Chung, 2022). Our study applied a larger cohort to comprehensively investigate the relationship between MR and motor, cognitive, and even non-motor symptoms. We found baseline MR was associated with MDS-UPDRS Parts I, II, III, MOCA, LNS, SDMT, SFT, ADL, REM, GDS, STAI and SCOPA overtime. These results suggest that the MR may be a comprehensive biomarker that reflecting an individual’s capacity to tolerate PD-related pathology, rather than merely representing motor deficit. Previous neuroimaging studies explored the brain structures relevant to MR in PD, reporting that both the basal ganglia and extra basal ganglia brain structures (including frontal and temporal lobes, limbic structure, nucleus accumbens, and thalamus) are related to MR in PD (Jeong, 2022, Youn, 2023). Besides, the motor reserve-related network has been shown to involve the bilateral basal ganglia (putamen, caudate, and globus pallidus), inferior frontal cortex, insula, amygdala, hippocampus, and cerebellar vermis, which play important roles in maintain normal brain function (Chung, 2020, Kim, 2022). These results demonstrate that the MR related brain structures contained substrates not only related to motor control, but also related to cognitive and emotional functions. The neuroanatomical relevance reinforces the robustness of our findings of the relationship between MR and motor and non-motor symptoms. Further studies are needed to elucidate the underling neural mechanisms contributing to different levels of MR.

Furthermore, we found PD patients with different MR exhibited different rates of motor symptom decline. Notably, our interaction results showed that PD patients with higher MR had a faster rate of motor progression, which appears contrary to our hypothesis. The concept of MR was initially proposed to reflect an individual's ability to resist pathological damage, with the expectation that higher MR would be associated with a slower progression of clinical symptoms. We speculate that the following reasons might explain the findings:(1) The influence of different stages of PD. Previous studies on cognitive reserve in AD spectrum have found that cognitive reserve is associated with slower cognitive decline; however, once the onset of cognitive dysfunction, AD patients with higher cognitive reserve may exhibit a more rapid cognitive decline (van Loenhoud, 2019). Our finding in the relationship between MR and motor progression was similar with that relationship between cognitive reserve and cognitive decline in AD. A previous study investigated the MR associated brain structure and found that a larger MR-associated striatal volume was associated with a lower requirement of LEED initially, but an subsequent accelerated LEED increment (Jeong, 2022). The clinical progression of PD may exhibit non-linearity (Zhao, 2010), and future exploration of the clinical progression rates of PD patients with varying motor reserves at different disease stages is needed to clarify the relationship between motor reserve and clinical outcomes in PD. (2) The ceiling effect of MDS-UPDRS scale. Our data show that PD patients in the low MR group have relatively high MDS-UPDRS III scores, indicating they are in a more advanced stage of the disease. Due to the ceiling effect of the scale, they may present relative smaller increase in MDS-UPDRS III scores during follow-ups, resulting in a slower rate of motor progression. Therefore, when assessing the progression of PD, it is necessary to observe not only the changes in motor scores but also the occurrence of clinically significant milestones. In our survival analyses of clinically significant milestones, we found that PD patients with lower MR had a more rapid progression to postural instability. Previous studies have shown that PD patients with higher MR proxies (higher education, dominant-side laterality, and active physical activity) had less severe motor deficits and exhibited a lower risk of reaching HY 3 stage (Lee, 2019). Our study, used residual approach to directly quantify the MR, can better illustrate the influence of MR on disease progression. Recently, Chung et. al investigated the association between initial level of MR and the risk of developing freezing of gait and levodopa-induced dyskinesia, reporting that greater MR were associated with lower risk for these clinical milestones (Chung, 2020). With a longer follow-up period, our study further demonstrated that PD patients with lower MR had higher risk of developing postural instability.

In this study we also investigated the associations between MR and the progression of non-motor symptoms. There was a trend that PD patients with lower baseline MR experienced a faster cognitive decline rate and PD patients with lower baseline MR had a higher risk of cognitive transition. Increasing evidence supports the link between cognitive dysfunction and motor disability in PD patients. Our study, based longer follow-up period and more comprehensive motor and non-motor evaluations, suggests that MR was not only a factor related to motor prognosis but a comprehensive indicator associated with the individual’s capacity to cope with neurodegenerative process in PD as well as comprehensive prognosis. Prior studies have reported that baseline motor symptoms and DAT binding were related disease prognosis (Brumm, 2023, Ravina, 2012). Our study focused on the MR estimate, derived from MDS-UPDRS motor scores and DAT, which may provide additional information for disease prognosis.

There are several limitations to this study. First. the individual MR were assessed by using the residual model with DAT availability in the posterior putamen and motor deficit. The DAT availability in posterior putamen can be downregulated as a compensatory mechanism to maintain synaptic dopamine levels in PD and may not purely reflect the nigrostriatal dopaminergic degeneration. Nevertheless, DAT imaging is still a widely used imaging modality for assessing nigrostriatal dopaminergic degeneration. Second the MR, assessed by using the residual model with general linear model, are inevitably correlated with the dependent variables (MDS-UPDRS III scores). However, we found the association between baseline MR and the longitudinal changes in motor and non-motor symptoms keep consistent after adjusting for the baseline MDS-UPDRS motor scores as a covariate. Our findings suggest that the MR is a more comprehensive index reflecting individual’s capacity to tolerate PD-related pathologies. Additionally, our survival analysis on gait instability was based on the definition of gait instability according to the H&Y scale, which may be somewhat imprecise. Future research should incorporate more specific gait-related scales. In this study, we also analyzed the occurrence of future 'walking and balance' milestones using both the MDS-UPDRS subscale and the H&Y scale, and the results were consistent with those obtained from the H&Y scale (see Supplementary Materials).

In conclusion, higher baseline MR was associated with better motor, cognitive and non-motor function in PD patients. The baseline MR was associated with the rate of decline for motor function. PD patients with lower MR had higher risk of postural instability and progression to MCI or dementia. Our study suggests that the MR can serve as a potential biomarker to monitor both motor and non-motor symptoms. Additional researches are needed to validate our findings and to reveal the exact mechanisms underlying variations in MR.

## CRediT authorship contribution statement

**Xueqin Bai:** Writing – review & editing, Writing – original draft, Visualization, Validation, Supervision, Software, Resources, Project administration, Methodology, Investigation, Funding acquisition, Formal analysis, Data curation, Conceptualization. **Shiwei Zhang:** Writing – review & editing, Writing – original draft, Visualization, Validation, Supervision, Software, Resources, Project administration, Methodology, Investigation, Formal analysis, Data curation. **Qiuyue Li:** Writing – review & editing, Writing – original draft, Visualization, Validation, Supervision, Software, Resources, Project administration, Methodology, Investigation, Formal analysis, Data curation. **Tao Guo:** Writing – review & editing, Writing – original draft, Visualization, Validation, Supervision, Software, Resources, Project administration, Methodology, Investigation, Funding acquisition, Formal analysis. **Xiaojun Guan:** Writing – review & editing, Writing – original draft, Validation, Supervision, Resources, Investigation, Formal analysis. **Andan Qian:** Writing – review & editing, Writing – original draft, Validation, Supervision, Resources, Methodology, Investigation, Formal analysis. **Shuangli Chen:** Writing – review & editing, Writing – original draft, Validation, Software, Resources, Methodology, Investigation. **Ronghui Zhou:** Writing – review & editing, Writing – original draft, Supervision, Project administration, Methodology, Investigation. **Yitong Cheng:** Writing – review & editing, Software, Methodology, Investigation. **Haoxin Chen:** Writing – review & editing, Supervision, Software, Methodology. **Zhaoke Gou:** Writing – review & editing, Software, Project administration, Methodology. **Chenglong Xie:** Writing – review & editing, Writing – original draft, Resources, Methodology, Investigation. **Zhen Wang:** Writing – review & editing, Writing – original draft, Visualization, Validation, Software, Resources, Methodology, Investigation, Funding acquisition. **Minming Zhang:** Writing – review & editing, Writing – original draft, Visualization, Validation, Supervision, Software, Resources, Project administration, Methodology, Investigation, Funding acquisition, Formal analysis, Data curation, Conceptualization. **Xiangwu Zheng:** Writing – review & editing, Writing – original draft, Visualization, Validation, Supervision, Software, Resources, Project administration, Methodology, Investigation, Funding acquisition, Formal analysis, Data curation, Conceptualization. **Meihao Wang:** Writing – review & editing, Writing – original draft, Visualization, Validation, Supervision, Software, Resources, Project administration, Methodology, Investigation, Funding acquisition, Formal analysis, Data curation, Conceptualization.

## Declaration of competing interest

The authors declare that they have no known competing financial interests or personal relationships that could have appeared to influence the work reported in this paper.

## Data Availability

Data will be made available on request.
