# Supplementary material for: The association of motor reserve and clinical progression in Parkinson’s disease
Source: Neuroimage Clin. 2024 Nov 9;44:103704. doi: 10.1016/j.nicl.2024.103704 (PMC11605422; doi:10.1016/j.nicl.2024.103704)
Supplement: Supplementary Data 1 [file mmc1.docx]

**Supplementary materials**

Table S1.The longitudinal changes of clinical characteristics.

| Variable | LME model^a^ | | Adjusted model^b^ | |
| --- | --- | --- | --- | --- |
|  | Estimate | *p* value | Estimate | *p* value |
| MDS-UPDRS I | 0.53 | **<0.001** | 0.53 | **<0.001** |
| MDS-UPDRS II | 0.96 | **<0.001** | 0.99 | **<0.001** |
| MDS-UPDRS III | 2.04 | **<0.001** | 2.05 | **<0.001** |
| ADL | -1.50 | **<0.001** | -1.51 | **<0.001** |
| REM | 0.12 | **<0.001** | 0.12 | **<0.001** |
| GDS | 0.10 | **<0.001** | 0.10 | **<0.001** |
| STAI | 0.41 | **0.015** | 0.27 | **0.07** |
| SCOPA | 0.39 | **<0.001** | 0.49 | **<0.001** |
| MOCA | -0.08 | **0.007** | -0.12 | **<0.001** |
| BJLOT | -0.04 | **0.043** | -0.06 | **<0.001** |
| HVLT | -0.13 | **<0.001** | -0.19 | **<0.001** |
| LNS | -0.10 | **<0.001** | -0.19 | **<0.001** |
| SDMT | -0.55 | **<0.001** | -0.97 | **<0.001** |
| SFT | -0.19 | **<0.001** | -0.32 | **<0.001** |

^a^ Covariates: age, sex, education, and LEDD

^b^ Covariates: age, sex, education, LEDD and baseline clinical score

MDS-UPDRS: Movement Disorders Society Unifed Parkinson’s Disease Rating Scale; ADL: Modified Schwab and England Activities of Daily Living; REM:Rapid eye movement Sleep Behavior Disorder Questionnaire; GDS: Geriatric Depression Scale; STAI: State-trait anxiety inventory; SCOPA: Scale for Outcomes in Parkinson’s disease-Autonomic; MoCA: Montreal Cognitive Assessment; BJLOT: Benton Judgment of Line Orientation Test; HVLT: Hopkins Verbal Learning Test for delayed recall; LNS: letter-number sequencing. SDMT: Symbol Digit Modalities Test; SFT: Semantic Verbal Fluency Test.

Table S2. Summary of linear mixed-effects models of the association between the baseline MR and clinical symptoms overtime and the effect of baseline MR on the progression in clinical symptoms using disease duration as the time scale, with age, sex, education, LEDD and baseline motor scores as covariates.

| Variable | baseline MR | basline MR *×* disease durations |
| --- | --- | --- |
|  | *p* value | *p* value |
| MDS-UPDRS I | **0.008** | 0.787 |
| MDS-UPDRS II | **< 0.001** | 0.231 |
| MDS-UPDRS III | **< 0.001** | **< 0.001** |
| ADL | **< 0.001** | 0.399 |
| REM | **0.014** | 0.631 |
| GDS | **0.002** | **0.014** |
| STAI | **< 0.001** | 0.202 |
| SCOPA | **0.002** | 0.520 |
| MOCA | **0.017** | **0.028** |
| BJLOT | 0.071 | **0.041** |
| HVLT | 0.081 | 0.408 |
| LNS | **0.012** | 0.397 |
| SDMT | **0.004** | 0.276 |
| SFT | **0.036** | 0.734 |

MDS-UPDRS: Movement Disorders Society Unifed Parkinson’s Disease Rating Scale; ADL: Modified Schwab and England Activities of Daily Living; REM:Rapid eye movement Sleep Behavior Disorder Questionnaire; GDS: Geriatric Depression Scale; STAI: State-trait anxiety inventory; SCOPA: Scale for Outcomes in Parkinson’s disease-Autonomic; MoCA: Montreal Cognitive Assessment; BJLOT: Benton Judgment of Line Orientation Test; HVLT: Hopkins Verbal Learning Test for delayed recall; LNS: letter-number sequencing. SDMT: Symbol Digit Modalities Test; SFT: Semantic Verbal Fluency Test.

The Kaplan-Meier survival analyses were performed to compare the cumulative probability of progression milestone in the "walking and balance" domain between the high MR and low MR groups during follow-up. The "walking and balance" domain milestone^[1]^ were based a comprehensive analysis of the sub-scores of the MDS-UPDRS scale and HY scale. The milestone event was achieved if any of the following criteria were met during the follow-up period as shown below.

| “Walking and balance”Progression milestone | Assessment | Criteria |
| --- | --- | --- |
| Walking and balance | MDS-UPDRS item 2.12 | Response ≥ 3 |
| Freezing | MDS-UPDRS item 2.13 | Response ≥ 3 |
| Gait | MDS-UPDRS item 3.10 | Response ≥ 3(ON or OFF) |
| Freezing of gait | MDS-UPDRS item 3.11 | Response = 4(ON or OFF) |
| Postural instability | MDS-UPDRS item 3.12 | Response ≥ 3(ON or OFF) |
| Hoehn and Yahr stage | Hoehn and Yahr Stage | Response ≥ 4(ON or OFF) |

We found that patients with low MR had a more rapid progression to "walking and balance" milestone compared to those with high MR (*p* = 0.02).


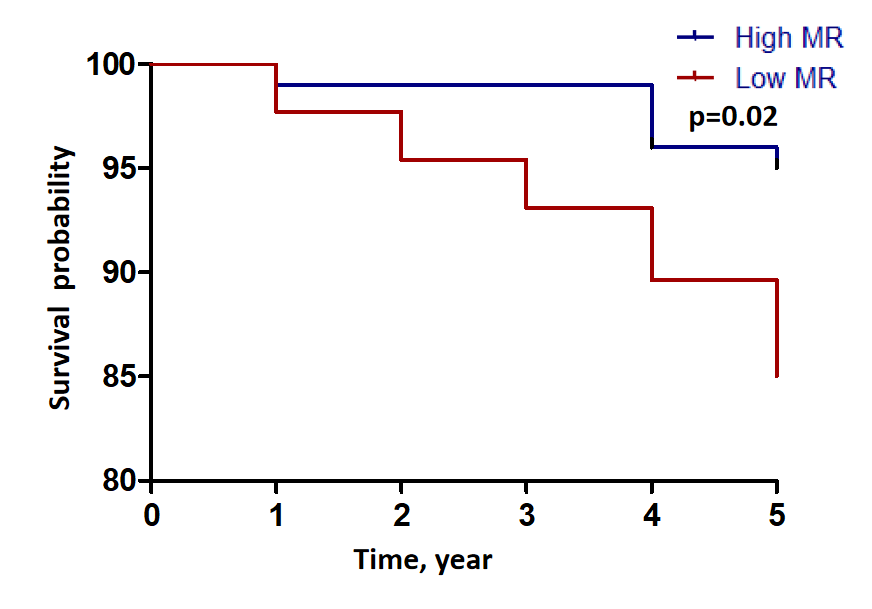


Figure 1. Kaplan-Meier curves of progression-free survival, as defined by reaching "walking and balance" milestone.

**Reference**

[1] Brumm M C, Siderowf A, Simuni T, Burghardt E, Choi S H, Caspell-Garcia C, Chahine L M, Mollenhauer B, Foroud T, Galasko D, Merchant K, Arnedo V, Hutten S J, O'Grady A N, Poston K L, Tanner C M, Weintraub D, Kieburtz K, Marek K, Coffey C S, Parkinson's Progression Markers I. Parkinson's Progression Markers Initiative: A Milestone-Based Strategy to Monitor Parkinson's Disease Progression [J]. J Parkinsons Dis, 2023, 13(6): 899-916.
